# Suppression of human metapneumovirus (HMPV) infection by the innate sensing gene CEACAM1

**DOI:** 10.18632/oncotarget.11979

**Published:** 2016-09-12

**Authors:** Mohammad Diab, Alon Vitenshtein, Yaron Drori, Rachel Yamin, Oded Danziger, Rachel Zamostiano, Michal Mandelboim, Eran Bacharach, Ofer Mandelboim

**Affiliations:** ^1^ The Lautenberg Center for General and Tumor Immunology, The BioMedical Research Institute Israel Canada of the Faculty of Medicine, The Hebrew University Hadassah Medical School, Jerusalem, Israel; ^2^ Central Virology Laboratory, Ministry of Health, Public Health Services, Chaim, Sheba Medical Center, Tel Hashomer, Ramat-Gan, Israel; ^3^ Department of Epidemiology and Preventive Medicine, School of Public Health, Sackler Faculty of Medicine, Tel-Aviv University, Tel-Aviv, Israel; ^4^ Department of Cell Research and Immunology, Faculty of Life Sciences, Tel Aviv University, Tel Aviv, Israel

**Keywords:** HMPV, CEACAM1, PRRs, PAMPs, RLRs, Immunology and Microbiology Section, Immune response, Immunity

## Abstract

The innate sensing system is equipped with PRRs specialized in recognizing molecular structures (PAMPs) of various pathogens. This leads to the induction of anti-viral genes and inhibition of virus growth. Human Metapneumovirus (HMPV) is a major respiratory virus that causes an upper and lower respiratory tract infection in children. In this study we show that upon HMPV infection, the innate sensing system detects the viral RNA through the RIG-I sensor leading to induction of CEACAM1 expression. We further show that CEACAM1 is induced via binding of IRF3 to the CEACAM1 promoter. We demonstrate that induction of CEACAM1 suppresses the viral loads via inhibition of the translation machinery in the infected cells in an SHP2-dependent manner. In summary, we show here that HMPV-infected cells upregulates CEACAM1 to restrict HMPV infection.

## INTRODUCTION

HMPV, discovered in 2001, is classified as the first human member of the *Metapneumovirus* genus of the paramyxovirus family [1]. HMPV is a ubiquitous respiratory pathogen, which is known to have been circulating in human populations for decades [1]. Several studies showed that HMPV is a leading cause of upper and lower respiratory tract infections in children and immunocompromised patients [2–12]. The clinical symptoms of patients infected with HMPV range from mild symptoms to severe bronchiolitis and pneumonia [5, 13–15], which can lead to death [16, 17].

Viruses contain conserved molecular structures known as PAMPs, which are recognized by several families of innate receptors, collectively named PRRs [18–23]. One important class of such receptors are the retinoic acid-inducible gene (RIG)-I-like receptors (RLRs) [18, 23, 24]. RLRs are intracellular sensors of viral components that include single stranded RNA (ssRNA) and double stranded RNA (dsRNA). Upon recognition of such PAMPs by the PRRs, different signaling pathways are activated leading to transcription of a multitude of antiviral genes [18, 24].

In epithelial cells, the innate immune sensor RIG-I was shown to sense HMPV infection leading to the secretion of type I IFNs through interferon response factor 7 (IRF7) and interferon response factor 3 (IRF3) [25–28]. Inhibition of RIG-I expression significantly decreases the production of type I IFN, pro-inflammatory cytokines and chemokines [29].

Carcinoembryonic antigen-related cell adhesion molecule 1 (CEACAM1) belongs to the carcino-embryonic antigen (CEA) family [30, 31]. It binds homophilically or heterophilically to members of the CEACAM family [32]. CEACAM1 is primarily an inhibitory receptor which delivers its inhibitory signal via tyrosine based inhibitory motifs (ITIMs), through the tyrosine phosphatase (SHP1) in immune cells and (SHP2) in non-immune cells [32–36]. In the current study, we show that following HMPV infection, CEACAM1 is induced by the innate sensing system to control viral production in an SHP2-dependent manner.

## RESULTS

### HMPV infection induces cell surface expression of CEACAM1

**Figure 1:**
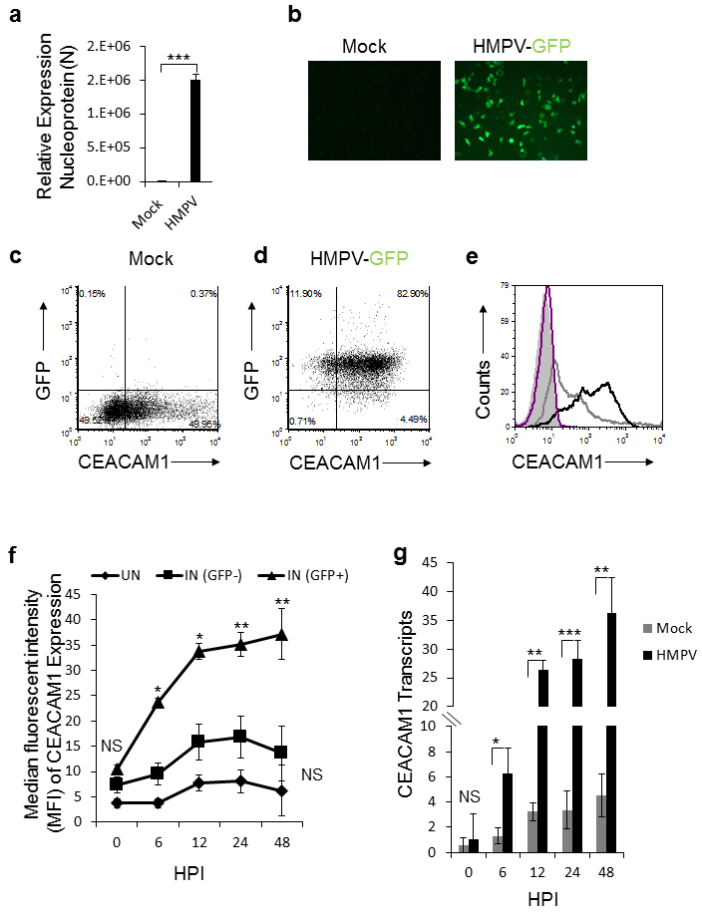
Expression of CEACAM1 in A549 cell line following HMPV infection **a.** qRT-PCR of the HMPV nucleoprotein (N) gene of HMPV in A549 infected cells (HMPV). The expression of nucleoprotein N in HMPV infected cells was determined relative to mock treated cells (Mock), whose expression level was set as 1. **b.** Fluorescent microscopy of mock treated (Mock) and A549 cells infected with the recombinant HMPV expressing GFP protein (HMPV-GFP). **c.-d.** Dot plots of CEACAM1 staining and GFP expression (indicative of virus infection) of mock (c) and HMPV-infected A549 cells (d). **e.** FACS analysis of CEACAM1 expression shown in (c and d) on the mock treated A549 cells (empty gray histogram) and on HMPV-infected A549 cells (empty black histogram), at 48h post infection. The filled gray histogram and the empty purple histogram represent the staining of the mock treated and infected A549 cells with control antibody, respectively. **f.** The expression of CEACAM1 was monitored on infected cells (IN (GFP+), on the uninfected cells that were present in the same culture IN (GFP-) and on parental A549 cells that were mock-treated (UN). The expression of CEACAM1 is presented as median fluorescent intensity (MFI) and is shown at various hours post infection (HPI). **g.** Real time PCR quantification of CEACAM1 expression on the mock-treated (Mock) and the HMPV infected cells (HMPV) during the indicated time points (HPI). NS, nonsignificant. Values are shown as means ± SEM. The figure shows data from two experiments combined. ***p <0.01,**p <0.03, *p < 0.05.

To investigate whether HPMV infection affects the expression of various natural killer (NK) cell ligands, we infected A549 cells with HMPV. 48 hours after the infection we validated that the cells were indeed infected by using qRT-PCR (Figure 1a) or by infection with recombinant HMPV virus expressing green fluorescent protein GFP (HMPV/GFP) [41] (Figure 1b). We next stained the mocked-infected and the infected cells for the expression of various immune ligands such as CEACAM1 (Figure 1c and 1d, respectively). Practically 100% of the cells were infected, as indicated in Figure 1d. We observed a significant induction of CEACAM1 expression following HMPV infection (Figure 1d and 1e). Analysis of expression kinetics of CEACAM1 showed that protein expression was visible as early as 6 hours post infection, peaking at 12 hours post infection (Figure 1f). Elevation of CEACAM1 mRNA was also noticed following HMPV infection (Figure 1g).

**Figure 2:**
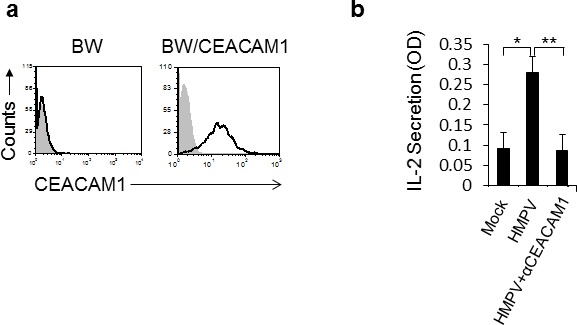
The expression of CEACAM1 on HMPV-infected A549 cells is functional **a.** FACS analysis of parental BW cells and BW cells transfected to expressed a chimeric protein composed of the extracellular portion of CEACAM1 fused to mouse zeta chain (BW/CEACAM1). The filled gray histogram is the background staining and the empty black histogram is the staining of CEACAM1. **b.** IL-2 secretion from the BW/CEACAM1 cells following 48h incubation with the indicated A549 cells that were either mock infected (Mock), infected with HMPV (HMPV) or infected with HMPV and blocked with anti CEACAM1 mAb (HMPV+αCEACAM1). Values are shown as means ± SEM. The figure shows data from three experiments combined. *p <0.05, **p < 0.02.

To test whether the HMPV-mediated CEACAM1 induction has functional consequences we used a cell-based reporter assay that employs the murine BW thymoma cells. For this assay, the BW cells were transfected with a construct that is composed of a chimeric protein in which the extracellular portion of CEACAM1 receptor is fused to the transmembrane and tail domain of the CD3z chain (Figure 2a). This system triggers IL-2 secretion upon binding of CEACAM1 to its ligand (which is CEACAM1). We infected A549 cells with HMPV and 48 hours later, we incubated the infected cells with BW/CEACAM1. As can be seen, significant induction of IL-2 secretion was observed following HMPV infection (Figure 2b). Blocking of the CEACAM1 interaction abrogated this induction, indicating that the induced IL-2 secretion resulted from homophilic CEACAM1-CEACAM1 interaction (Figure 2b).

### CEACAM1 expression is induced by the innate immune cellular RIGI-IRF3 sensing system

**Figure 3:**
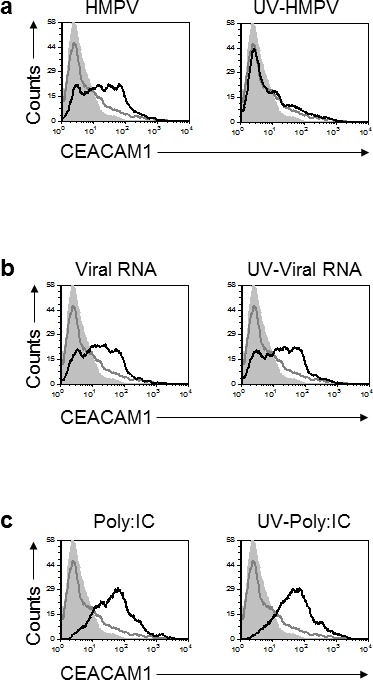
Induction of CEACAM1 on A549 cells following RNA treatments **a.** Analysis of CEACAM1 expression on A549 cells, 3 days post infection. The A549 cells were infected either with HMPV (left) or with UV-inactivated virus (right, UV-HMPV). **b.** A549 cells were transfected with HMPV RNA (Viral RNA) or with HMPV RNA that underwent UV inactivation (UV-Viral RNA). (c) A549 cells were transfected either with Poly:IC (left) or with Poly:IC that was inactivated by UV treatment (UV-Poly:IC). For all figure parts CEACAM1 staining of the treated cells is represented by the black empty histograms. The background staining is represented by the gray filled histograms and mock CEACAM1 staining is the gray empty histograms. The background staining and the CEACAM1 staining of the MOCK cells is identical in all figure parts **a.-c.** and is shown several times for clarity. The background staining of the various treatment was similar to the MOCK treatment and is not shown in the figure. Figure shows one representative experiment out of two independent experiments.

We next proceeded to study the mechanism of HMPV-mediated induction of CEACAM1. Initially, we observed that a viable virus is required to induce CEACAM1 expression, since CEACAM1 was not induced following infection with UV-inactivated virus (Figure 3a). To investigate whether HMPV RNA can induce CEACAM1 expression we isolated the HMPV RNA and transduced it to A549 cells. As can be seen in Figure 3b, viral RNA, UV-treated or not, induced the expression of CEACAM1. Finally, we investigated whether the RNA-mediated induction of CEACAM1 is specific to the viral RNA only. For this we transfected the A549 cells with Polyinosinic-polycytidylic acid (PolyI:C), a synthetic analog of double-stranded RNA and observed that PolyI:C transfection, UV-treated or not, also induce CEACAM1 expression (Figure 3c). These observations suggest that the cellular nucleic acid sensing system is responsible for the induction of CEACAM1.

**Figure 4:**
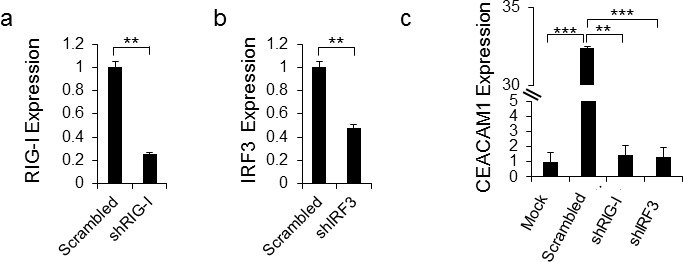
CEACAM1 induction is mediated by RIG-I and IRF3 **a.-b.** qRT-PCR quantification of RIG-I (a) and IRF3 (b) expression in A549 cells that underwent IRF3 and RIG-I targeted shRNA knockdowns compared with the same cells transduced with scrambled shRNA (Scrambled set as 1). **c.** The scrambled A549 cells and the specific shRNA knockdown cells from (a) and (b) were infected with hMPV and analyzed by FACS for induction of CEACAM1 on mock (Mock) cells was set up to be 1. Figure shows one representative result out of three independent experiments. **P < 0.01, ***P < 0.001.

Previous studies have shown that RIG-I is the sensor of HMPV RNA [26]. We next tested whether RIG-I, via its mediator IRF-3, is responsible for the CEACAM1 induction. For this, we knocked-down RIG-I (Figure 4a) and IRF3 (Figure 4b) by using short hairpin RNAs (shRNAs). As can be seen, knockdowns of both proteins almost completely abolished the upregulation of CEACAM1 following 48 hours of HMPV infection, compared to the control (Figure 4c). This finding demonstrates that the innate immune RNA sensor, RIG-I, leads to the induction of CEACAM1 through the RIGI-IRF3 pathway.

**Figure 5:**
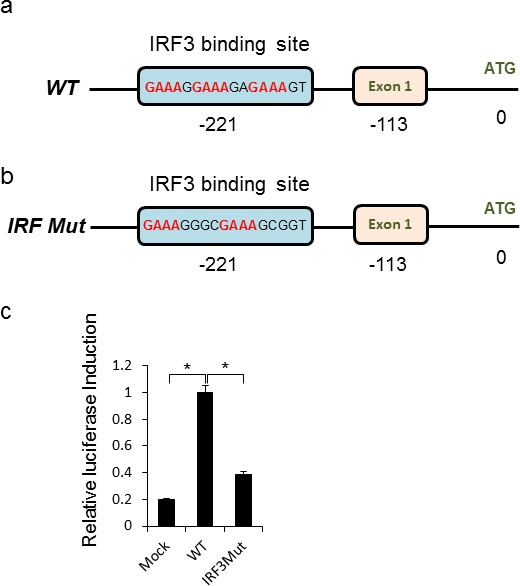
Direct IRF3 mediated induction of CEACAM1 during HMPV infection **a.-b.** Schematic representation of the CEACAM1 promoter region with “Exon1” indicating transcript initiation point and “ATG” the start of the CEACAM1 open reading frame (ORF). The wild-type IRF3 binding site sequence is indicated (a) along with the mutation introduced into the IRF3 site (IRF3 Mut, b). **c.** Fold increase in luciferase activity in A549 cells. Mock or HMPV infected A549 cells were transfected with luciferase encoding vector fused downstream to the wild-type CEACAM1 promoter (WT), or luciferase fused to the mutated CEACAM1 promoter (*I*RF3M). The figure shows data from three experiments combined. Statistical analysis was calculated on the data from all experiments combined. *p < 0.03.

The *CEACAM1* promoter contains a predicted IRF3 binding site (Figure 5). To test whether this site mediated the CEACAM1 induction during HMPV infection, we used a luciferase reporter assays in which 600bp of the wild-type or IRF3 binding site mutated (IRF3 Mut) promoter sequences was fused to luciferase (Figure 5a and 5b respectively). While the CEACAM1 promoter sequence mediated a strong induction of luciferase activity, following 48 hours of HMPV infection, mutation in the predicted IRF3 binding site, significantly decreased the luciferase activity (Figure 5c).

### CEACAM1 suppresses HMPV virus production by inhibiting protein synthesis in an SHP2-dependent manner

**Figure 6:**
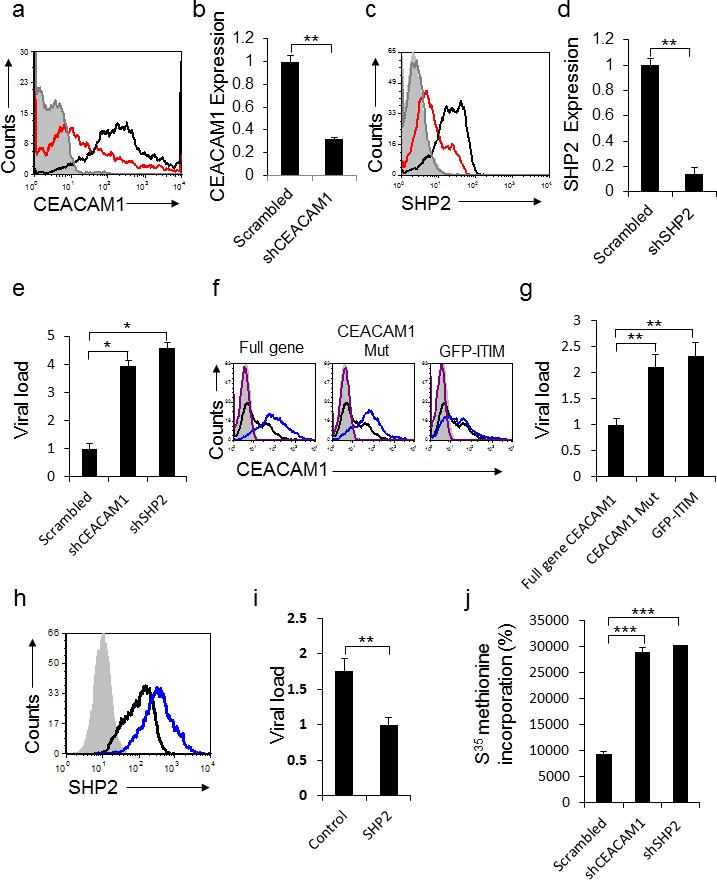
CEACAM1 inhibits HMPV infection through SHP2 **a.** CEACAM1 was knockdown in A549 cells using specific shRNA (red histogram), scrambled shRNA was used as control (black empty histogram). The gray empty histogram and the gray filled histogram represent the staining with control Ab of A549 cells transduced with shRNA against CEACAM1 and scrambled shRNA, respectively. **b.** qRT-PCR quantification of CEACAM1 down regulation following the shRNA knockdown. A549 cells transduced with scrambled was set up as 1. **c.** SHP2 was knockdown on A549 cells using specific shRNA (red histogram), scrambled shRNA was used as control (black empty histogram). The gray empty histogram and the gray filled histogram represent the staining with control Ab of A549 cells transduced with shRNA against SHP2 and scrambled shRNA, respectively. **d.** Real time PCR quantification of SHP2 down regulation following the shRNA knockdown of SHP2. A549 cells transduced with Scrambled was set up as 1. **e.** Quantification of viral loads in the supernatants of A549 infected cells expressing CEACAM1 or SHP2 specific shRNAs compared to the scrambled (Scrambled, set as 1). **f.** FACS analysis of the overexpressed full CEACAM1 gene, CEACAM1 lacking the intracellular domain (CEACAM1 Mut) or a construct contain the intracellular domain fused to GFP (GFP-ITIM), showed as blue empty histograms. Control expression of CEACAM1 on untreated A549 cells (black empty histogram). The purple empty histogram and the gray filled histogram represent the staining with control Ab of A549 cells overexpressed with full gene CEACAM1or CEACAM1 mutants and untreated cells, respectively. **g.** Quantification of viral loads in the supernatants of A549 infected cells expressing the full gene of CEACAM1, (CEACAM1 Mut) or (GFP-ITIM). The full gene CEACAM1, set as 1. **h.** SHP2 overexpression in A549 cells (blue empty histogram), as compared to control SHP2 expression (black empty histogram). The gray filled histogram represent the staining with control Ab of A549 cells over expressing with SHP2. The control staining of parental cells was similar and is not shown in the figure. **i.** Quantification of viral loads in the supernatants of A549 infected cells overexpressing SHP2 compared to the control. The overexpressed SHP2, set as 1. **j.** A549 cells stably expressing shCEACAM1 or shSHP2 were analyzed for total protein production by assessing the rate of ^35^S-Methionine incorporation compared to the scrambled. The figure shows data from two experiments combined. *P < 0.05; **P < 0.01; ***P < 0.001.

We next proceeded to understand why CEACAM1 expression is induced following HMPV infection. Homophilic binding of CEACAM1 transmits an inhibitory signal via the SHP2 phosphatase in non-immune cells. We initially knocked-down either CEACAM1 (Figure 6a, qRT-PCR verification in 6b) or SHP2 (Figure 6c qRT-PCR verification in 6d), in A549 cells by using specific shRNAs. Twenty four hours following HMPV infection, viral loads were quantified. As can be seen, a significant elevation in viral loads was observed in cells expressing the CEACAM1 and SHP2 specific shRNAs compared to the control (Figure 6e). To further corroborate these results we overexpressed the full CEACAM1 gene or two mutants of CEACAM1: 1) CEACAM1 lacking the intracellular ITIM containing signaling domain (CEACAM1 Mut) and 2) Fusion of the intracellular signaling domain to GFP (GFP-ITIM) (Figure 6f). Following 24 hours of HMPV infection, significant elevation of viral loads was observed in cells expressing CEACAM1 Mut or GFP-ITIM, as compared to cells expressing the full length CEACAM1 gene (Figure 6g). Additionally, we over-expressed the SHP2 phosphatase in A549 cells (Figure 6h). Following 24 hours of HMPV infection, we observed a significant decrease in viral loads in cells over-expressing SHP2 compared to control (Figure 6i). Finally, we proceeded to investigate the mechanism by which CEACAM1 and its phosphatase SHP2 inhibit the HMPV production. We assessed protein production in A549 cells expressing shRNAs against CEACAM1 and SHP2. The treated cells were labeled with ^35^S-Methionine and following 24 hours of HMPV infection the ^35^S-Methionine labelling was determined. As can be seen, cells expressing shRNAs targeting CEACAM1 or SHP2, exhibited about a 3 fold increase in ^35^S-Methionine incorporation following infection, as compared to the scrambled shRNA (Figure 6j). Thus, we conclude that CEACAM1 suppresses HMPV infection in an SHP2-dependent manner by inhibiting the protein production machinery.

## DISCUSSION

We know today that practically all nucleated cells participate in innate recognition of viruses. They all contain cellular sensors (PRRs) that detect PAMPs, among them are viral nucleic acids. This leads to the production of IFNs, antiviral genes and activation of signaling cascades which results in pathogens inhibition [18–20, 24, 42].

**Figure 7:**
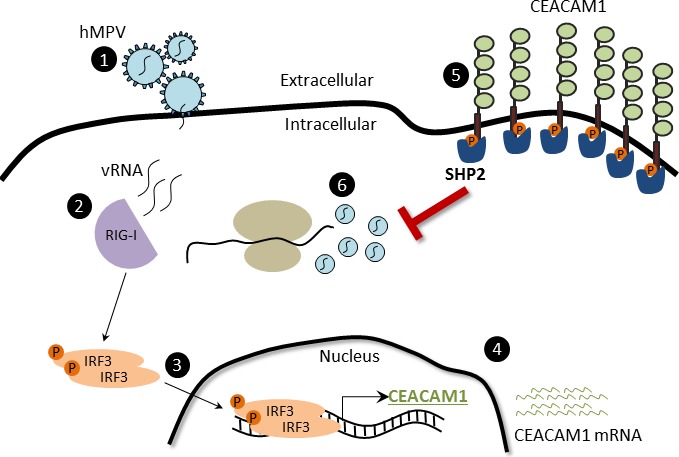
The CEACAM1-mechanism of action during hMPV infection (1) HMPV binds and infects the cells. (2) HMPV RNA is sense by RIG-I, which subsequently activates downstream the phosphorylation of IRF3. (3) IRF3 undergoes conformational changes and dimerization, then translocates to the nucleus and binds the CEACAM1 promoter. (4) CEACAM1 mRNA is transcribed. (5) CEACAM1 is overexpressed on the cell surface of infected cells. (6) The SHP2 phosphatase anchor on CEACAM1 ITIMs and subsequently inhibits the HMPV replication by inhibiting protein production.

We recently demonstrated that upon sensing of influenza by RIG-I and HCMV by IFI16, CEACAM1 expression is induced [43]. Here we show that RIG-I sensing of HMPV also leads to CEACAM1 induction and to the establishment of an anti-viral state in the infected cells. We suggest the following (Figure 7): 1) Upon HMPV infection, the negative single stranded RNA of HMPV is exposed in the cytoplasm. 2) RIG-I senses viral RNA 3) IRF3 is activated, and translocate into the nucleus where it binds the CEACAM1 promoter. 4) CEACAM1 transcription is induced. 5) CEACAM1 protein is expressed. 6) The hemophilic binding of CEACAM1 leads to activation of SHP2 phosphatase which in turns leads to inhibition in the cellular translation and to viral suppression.

The mechanism underlying the CEACAM1-mediated suppression involves inhibition of the cellular translation machinery, can be detrimental to healthy cells. However, we demonstrate that CEACAM1 induction is restricted to the HMPV-infected cells. We propose that the innate immune system developed this specific mechanism that harnesses CEACAM1 as an anti-viral mechanism, unique properties and due to its homophilic interactions. Thus, through the induction of CEACAM1 only, virus production is inhibited.

## MATERIALS AND METHODS

### Viruses and cell lines

Vero, kidney epithelial cells derived from an African green monkey, and A549, human alveolar type II-like epithelial cells, were maintained in Dulbecco's modified Eagle medium (DMEM) with standard supplements and 10% fetal calf serum (FCS). To propagate HMPV, around 70% confluent Vero cells were infected with recombinant (HMPV-GFP) strains as previously described [37] using MOI 0.1 in medium containing 0.25 mg/ml trypsin for one hour. DMEM medium with standard supplements containing 3% FCS was then added. Five days after infection, cells were scraped from the plates, collected, freeze-thawed (−80°C/37°C) three times and crude viruses were stored at -80°C. Viral loads were determined by using quantitative RT-PCR (qRT-PCR) and/or by using fluorescence microscopy. For A549 infection experiments, around 70% confluent cells were infected with HMPV, in serum-free media with 0.25 mg/ml trypsin for 1 hour incubation at 37°C, then maintained in DMEM medium with standard supplements containing 2% FCS.

### Viral RNA isolation and transfections

For preparation of shRNA, 293T cells were co-transfected with the lentiviral vector containing the shRNA, a plasmid encoding the lentiviral Gag/Pol, and a plasmid encoding the VSV-G at a 10:6.5:3.5 ratios respectively. Supernatants contaning the viral particles were collected after 48 hours. A549 cells were selected for puromycin resistance at 5μg/ml. Viral RNA that was transfected was isolated from cell free purified viral stocks by easyMag system (BioMerieux). Viral RNA transfection was performed on cells that were plated in 24 well plates, at 50K cells/well, which were subsequently transfected with 1μg/ml of DNA with 2μl/μg of LT-1 (MirusBio) transfection reagent per DNA, according to manufacturer's recommendations. UV inactivation prior to viral infection or viral RNA transfection, was performed with the UV Stratalinker 2400 (StrataGene) at 0.99 Joule.

### FACS staining

Staining for CEACAM1 was conducted by FACS staining for human CEACAM1 with anti-CEACAM1 mAb (Biolegend). Intracellular staining for SHP2 was conducted using an anti-SHP2 antibody (Santa Cruz). Intracellular FACS staining for SHP2 was based on methanol fixation protocol. Briefly, cells were perforated in methanol at -20°C, rehydrated in PBS for half an hour, and then stained with antibodies in 5% BSA in PBS at room temperature.

### Cloning, qRT-PCR and shRNAs

Stable transduction of CEACAM1, CEACAM1-Mut, GFP-ITIM and SHP2 transfectants was based on lentiviral and retroviral expression systems. For mRNA quantification, total RNA was isolated from cells using the Total RNA isolation kit (Zymoresearch). RNA was reverse transcribed with Moloney murine leukemia virus reverse transcriptase (Invitrogen) and with polyT primer (Sigma). Quantitative amplification was conducted on an ABI PRISM 7900 real-time PCR system (Applied Biosystems) with gene specific primers and Platinum SYBR Green qPCR SuperMix-UDG with ROX (Invitrogen). Newly synthesized transcript analysis was performed as previously described [38]. RNA was reverse transcribed and analyzed by real-time PCR. Primers used for the real-time PCR:

Amplification of hCEACAM1 long variant only fwd 5′TGCTGAACGTAAACTATAATGCTCT, rev 5′ GGAGACTGAGGGTTTGTGCT

Amplification of HMPV nucleoprotein gene (N) [39].

Forward (fwd) 5′ CATATAAGCATGCTATATTAAAAGAGTCTC, rev 5′ CCTATTTCTGCAGCATATTTGTAATCAG

IRF3 fwd 5′GATGCACAGCAGGAGGATTT, rev 5′ TAAACGCAACCCTTCTTTGC;

RIG-I fwd 5′ATCCCAGTGTATGAACAGCAG; rev 5′GCCTGTAACTCTATACCCATGTC.

SHP2 fwd GGGTGGAGACACGACACTTT; rev 5′

GGTTCTTCACCAAGCTGGAC

HPRT and GAPDH used as normalizers for qPCR analyses. HPRT fwd 5′TGACACTGGCAAAACAATGCA; rev 5′GGTCCTTTTCACCAGCAAGCT GAPDH fwd 5′TGCACCACCAACTGCTTA; rev 5′GGATGCAGGGATGATGTTC. Primers used for cloning and mutating CEACAM1 promoter (cloned with XhoI and HindIII) in pGL4.14 *firefly* luciferase expression vector (Promega) fwd 5′CGCCTCGAGCCTGGACTTGGGTCTCTGTC; Mutation reversed (rev) 5′ TCCTACCTTTGTC CTTACCGCTTTCGCCCTTTCTGTCTACATTTT; Mutation fwd 5′ AAAATGTAGACAGAA AGG CGAAAGCGGTAAGGACAAAGTAGGA; CEACAM1 promoter, rev 5′CGCAAGCTTTCACCTGTGGAGGAGAGCTT. shRNA sequences were based on the pLKO.1 lentiviral vector backbone with puromycin selection marker (Sigma).

IRF3

5′ CCGGGATCTGATTACCTTCACGGAACTCGAGTTCCGTGAAGGTAATCAGATCTTTTT.

RIG-I

5′CCGGCCATGTGAAGTACAAGACATTCTCGAGAATGTCTTGTACTTCACATGGTTTTTTG.

CEACAM1

5′CCGGCCACCTAACAAGATGAATGAACTCGAGTTCATTCATCTTGTTAGGTGGTTTTTG.

SHP2

5′CCGGGCAGTTAAATTGTGCGCTGTACTCGAGTACAGCGCACAATTTAACTGCTTTTT.

### BW assay

BW transfected cells were prepared as described elsewhere [40]. 50,000 of the appropriate BW or BW transfectants were incubated together with equal numbers of irradiated (6000 rad) mock-infected or HMPV-infected A549 cells for 48 hours at 37°C and 5% CO2. Following 48 hours incubation, the presence of mouse IL-2 in the supernatants was determined using standard enzyme-linked immunosorbent assay (ELISA). Student's t test was used to determine significant differences.

### Luciferase assay

Cells were grown to 50%-60% confluence in 24-well plates. Cells were then

transfected using LT-1 transfection reagent (MirusBio), with 60ng of the respective *firefly* luciferase reporter vector pGL4.14 and 5ng of control vector, encoding *Renilla* luciferase, pRL-CMV (Promega), in a final volume of 0.5 ml. *Firefly* and *Renilla* luciferase activities were then measured consecutively using the Dual-luciferase assays (Promega), 48 hr after transfection.

### Protein translation assay

A549 cells grown in 24-well replicates were resuspended in methinonine-free DMEM based (Sigma) medium. Following methionine starvation, labeled [^35^S] Methionine was supplemented to the medium to a final labeling concentration of 0.05μCi/μL for 5 hours of incubation. Cells were then washed with PBS, lysed in 1ml of NaOH 0.1M, supplemented with 1:3 ration of scintillation liquid and analyzed by β-Counter.

### Statistical analysis

Statistical significance was determined by Students t-test. P value of less than 0.05 was considered significant and indicated in figures and figure legends.
